# Perspectives of women living with type 1 diabetes regarding preconception and antenatal care: A qualitative evidence synthesis

**DOI:** 10.1111/hex.13876

**Published:** 2023-11-01

**Authors:** Ana Toledo‐Chavarri, Janet Delgado, Beatriz Rodríguez‐Martín

**Affiliations:** ^1^ Canary Islands Health Research Institute Foundation, (FIISC) Tenerife Spain; ^2^ The Spanish Network of Agencies for Health Technology Assessment and Services of the National Health System (RedETS) Madrid Spain; ^3^ Research Network on Health Services in Chronic Diseases (REDISSEC) Spain; ^4^ Network for Research on Chronicity, Primary Care, and Health Promotion (RICAPPS) Spain; ^5^ Department of Philosophy I University of Granada, Campus Universitario de Cartuja CP Granada Spain; ^6^ Department of Nursing, Physiotherapy and Occupational Therapy Faculty of Health Sciences, University of Castilla‐La Mancha Talavera de la Reina (Toledo) Spain

**Keywords:** preconception care, pregnancy, qualitative evidence synthesis, type 1 diabetes

## Abstract

**Introduction:**

Pregnant women with type 1 diabetes may have an increased risk of complications for both the baby and themselves. Educational programmes, preconception planning, strict glycemic control and comprehensive medical care are some of the antenatal interventions that have been proposed to improve the outcomes of pregnant women with type 1 diabetes. While some evidence‐based recommendations about antenatal care are included in clinical practice guidelines (CPGs), the views, and experiences of women with type 1 diabetes about these interventions are not well known.

**Aim:**

To understand and synthesize the perceptions of women with type 1 diabetes about the interventions before pregnancy.

**Method:**

A qualitative evidence synthesis (QES) was carried out with a framework analysis guided by the Cochrane Qualitative and Implementation Methods Group approach. Three online databases (Medline, Embase and Web of Science) were searched. We included qualitative articles that were published from 2011 to 2021 and which were available in English or Spanish.

**Findings:**

Ten references met the inclusion criteria of the study and were included. Three main themes were identified: (a) acceptability of antenatal care, (b) feasibility and implementation consideration and (c) equity and accessibility difficulties.

**Conclusion:**

Continuity of care, coordination between health professionals and services, and a more holistic approach are the key aspects women say need to be considered for more acceptable, feasible and equitable preconception and antenatal care.

**Patient or Public Contribution:**

This QES was carried out as part of the CPGs on diabetes mellitus type 1, carried out as part of the Spanish Network of Health Technology Assessment Agencies. In this CPG, the representatives of the patient associations are Francisco Javier Darias Yanes, from the Association for Diabetes of Tenerife, who has participated in all the phases of the CPG; Aureliano Ruiz Salmón and Julián Antonio González Hernández (representatives of the Spanish Diabetes Federation (FEDE) who have participated as collaborator and external reviewer, respectively.

## 1 INTRODUCTION

Women with type 1 diabetes mellitus (T1DM) can have healthy pregnancies, but they may experience additional challenges in managing their disease. Poor control of diabetes during pregnancy may lead to increased problems for the baby and the mother. Women with T1DM may have a higher risk of first‐trimester miscarriage, congenital anomalies of the baby, prematurity, prenatal mortality and pre‐eclampsia.^1^, ^2^, ^3^ If blood glucose levels are not well controlled, the newborn is at higher risk of hypoglycemia, hypocalcemia, hyperbilirubinemia and polycythemia.^2^, ^3^ In addition, women with T1DM present a high caesarean delivery rate.^4^ Therefore, pregnancy usually increases anxiety and stress for women with T1DM.^5^ Careful monitoring of blood glucose levels and detailed planning of daily activities are necessary for a healthy pregnancy. All this can cause women with diabetes to experience exaggerated feelings of responsibility and perceived demands on the part of the baby, which generates constant worry, guilt, fear and too much pressure to provide the best conditions to allow the birth of a healthy baby.^6^, ^7^, ^8^

Several interventions before the pregnancy have been developed to improve the outcomes of pregnant women with T1DM. Some educational interventions, preconception planning, strict glycemic control and comprehensive medical care can reduce maternal, foetal and pregnancy risks.^9^ In this regard, there are studies showing that improving preconception care for women with pre‐existing diabetes can diminish adverse outcomes.^10^, ^11^ The American Diabetes Association^12^, ^13^ estimates that preconception counselling can reduce the incidence of major congenital malformations from 9% to 2%. A recent systematic review^11^ concluded that preconception care for women with pregestational type 1 or type 2 diabetes mellitus is effective in decreasing congenital malformations, and improving the risk of preterm delivery and admission to the neonatal intensive care unit. In addition, this care probably reduces maternal HbA1C in the first trimester of pregnancy, perinatal mortality and the cases of small for gestational age births.^11^ However, although the impact of preconception counselling on cognitive, psychosocial and behavioural outcomes, as well as its cost‐effectiveness have been assessed for policy and implementation decision‐making, the perspectives of women with T1DM are considered less in the process. Women with T1DM are experts in their disease and self‐care, but at the same time, they are in a situation in need of care in the context of the challenges of pregnancy.^14^ A better understanding of their perspectives can play a crucial role in relation to the acceptability, feasibility and equitability of antenatal care interventions. This is of particular relevance when developing clinical practice guidelines (CPGs). CPGs are evidence‐based, clearly written and easily accessible to clinicians. However, well‐developed CPGs and effective CPG implementation methods are needed, as both development and implementation need to be improved to have a better impact in clinical practice. To improve the impact of the recommendations, an important aspect might be to include the patient's views, since patient and public involvement is considered an essential element of trustworthy guideline development.^15^ Thus, the goal of this article is to address what the women's perceptions are about the interventions before pregnancy that can be recommended for T1DM women. The research was carried out as part of the development of the CPG on diabetes mellitus type 1, funded by the Spanish Ministry of Health.

## 2 METHODS

A qualitative evidence synthesis (QES) was developed with a framework analysis guided by the Cochrane Qualitative and Implementation Methods Group approach.^16^ Enhancing transparency was used in reporting the synthesis of the qualitative research checklist (ENTREQ) to guide the reporting of this QES.^17^ which can be found in Supporting Information: File S1. The framework analysis used the categories of acceptability, feasibility and equity from the *Evidence to Decision framework* from *Grading of Recommendations Assessment, Development and Evaluation*.^18^, ^19^ This framework sets out research questions that can guide the comprehension of each category (Table 1).

**Table 1:** Frameworks research questions.

Research questions related to acceptability, feasibility and equity	
Acceptability Is the intervention acceptable to key actors?	Are there key stakeholders that would not accept the distribution of the benefits, harms and costs? Are there key stakeholders that would not accept the costs or undesirable effects in the short term for desirable effects (benefits) in the future? Are there key stakeholders that would not agree with the values attached to the desirable or undesirable effects (because of how they might be affected personally or because of their perceptions of the relative importance of the effects for others)? Would the intervention adversely affect people's autonomy? Are there key stakeholders that would disapprove of the intervention morally, for reasons other than its effects on people's autonomy (e.g., in relation to ethical principles such as no maleficence, beneficence or justice)?
Feasibility Is the intervention feasible to implement?	Is the intervention or option sustainable? Are there important barriers that are likely to limit the feasibility of implementing the intervention (option) or require consideration when implementing it?
Equity What would be the impact on equity?	Are there groups or settings that might be disadvantaged in relation to the problem or options that are considered? Are there plausible reasons for anticipating differences in the relative effectiveness of the option for disadvantaged groups or settings? Are there different baseline conditions across groups or settings that affect the absolute effectiveness of the intervention or the importance of the problem for disadvantaged groups or settings? Are there important considerations that should be made when implementing the intervention to ensure that inequities are reduced, if possible, and that they are not increased?

*Note*: Adapted from Moberg et al.^19^

A scoping search in Pubmed, CINHAL and PSYCINFO was performed to gain an overview of the existing literature. The following two search strategies were used: ‘type 1 diabetes mellitus and (preconception care or preconception intervention or prepregnancy care) and (qualitative or interview or focus group)’ and ‘type 1 diabetes mellitus and (preconception care or preconception intervention or pre‐pregnancy care) and (acceptability or feasibility or equity or ethics)’.

The scoping search helped refine and test a systematic search. Additionally, the scoping phase showed the necessity to include a more general and introductory theme that collected the experiences of T1DM women to contextualize the framework research questions.

The literature in Spanish and English was searched in Medline, Embase and Web of Science. The search was limited to the last 10 years (2011–2021). See Supporting Information: File S2 for the search strategies. References of the included studies were screened to find potential additions.

**Figure 1:**
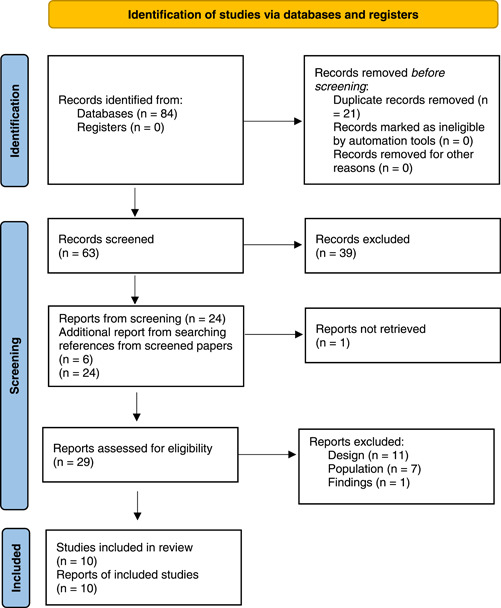
PRISMA flow diagram.^20^ For more information, visit: http://www.prisma-statement.org/. PRISMA, Preferred Reporting Items for Systematic Reviews and Meta‐Analyses.

Two researchers independently screened each reference for eligibility, first by title and abstract and then by reading the full text. References were included if they addressed the objectives of this review, used qualitative techniques and reported qualitative findings separately. Studies in languages other than Spanish and English were excluded. Disagreements were resolved by discussion within the team. The complete inclusion and exclusion criteria can be found in Table 2 and a Preferred Reporting Items for Systematic Reviews and Meta‐Analyses (PRISMA) (Figure 1).

**Table 2:** Inclusion and exclusion criteria for the selection of the studies.

	Population	Context	Findings	Design
Inclusion	People who are pregnant or want to become pregnant of any age and sex with a diagnosis of T1DM.	Pregnancy planning or pregnancy care in the health care system.	Experiences or trajectory of care. Acceptability, feasibility, equity or considerations for implementation of interventions.	Qualitative studies or mixed methods studies reporting results separately.
Exclusion	Other populations (diabetes type 2, gestational diabetes, etc.).	Any other DM1 care.	Any other finding.	Randomized clinical trials, nonrandomized clinical trials, quasi‐experimental studies, narrative reviews, editorials, letters to the editor and abstracts.

Abbreviation: T1DM, type 1 diabetes mellitus.

A thematic synthesis adapted from Thomas and Harden^21^ was carried out within the framework analysis using the software Nvivo12® to support the process. The whole team independently coded a sample of two studies each to develop an initial code book based on the abovementioned framework for this study extended with deductive codes from the included studies. The code book was then discussed within the team and used to code the rest of the studies. The final version of the code book can be seen in Table 3. Two independent reviewers extracted all relevant qualitative findings in each of the studies and descriptive themes were generated and discussed among the whole team. A data reporting form was prepared according to the selected framework and the cited categories of acceptability, feasibility and equity.

**Table 3:** Code book.

Category	Codes	Description
*Theme: Acceptability*		
Support	Professional support	Relationships and communication with health professionals.
	Informal support	Family and social support received during pregnancy planning and development.
Information	Health professionals' lack of information	Health professionals' lack of information.
	Needs and lack of information related to pregnancy	Preference for receiving information related to pregnancy and its risks.
	Prenatal advice/preconception care	Availability of services of prenatal advice/preconception care.
	T1DM and pregnancy education	Perceptions and experiences related to pregnancy and T1DM.
Empowerment	Autonomy in decision‐making	Autonomy in decision‐making in relation to pregnancy.
	Self‐efficacy	Pregnancy as a moment for life style changes and T1DM control improvement.
Pregnancy planning	Perception of pregnancy planning	Perceptions on and experiences with pregnancy planning.
	Experience sharing	Importance of experience sharing related to previous pregnancies with T1DM.
*Theme: Feasibility*		
Information needs	Information needs	Information needs of women with T1DM in relation to neonatal advice and pregnancy planning.
Person‐centred care	Communication	Communication with health professionals.
	Perceptions of model of care	Perception in relation to the model of care (holistic and individualized care).
	Trust	Trust and distrust in health professionals.
	Continuity of care	Coordination of health services. Perceptions in relation to continuity or advancement of care.
*Theme: Equity*		
Access	Barriers to access	Barriers to access to midwives and specialized care.
*Theme: Experiences*		
Experiences	Previous pregnancies	Experiences of previous pregnancies.
Feelings and beliefs	Anxiety and stress	Anxiety and stress in relation to pregnancy and maternity.
	Pressure and guilt	Feelings of pressure and guilt in relation to self‐care during pregnancy, constant worries in relation to the baby.
		Emotional burdens related to being advised against pregnancy by health care professionals.
		Frustration or resentment towards diabetes.
	Contradictions	Contradictions in relation to pregnancy and its social significance, fears.
	Control	Perceptions in relation to glycemic control.
	Acceptance	Feelings and beliefs related to acceptance of the disease, vulnerability and the impossibility of perfect control.
	Pregnancy and maternity beliefs	Beliefs about pregnancy and maternity that can impact conception planning and care.
Burden	Hard work	Pregnancy and diabetes described as hard work.
	Work	Impact on work of pregnancy planning and development.

Abbreviation: T1DM, type 1 diabetes mellitus.

The research team has some experience that influenced the analysis of the results of the QES. The team included mothers and a person with T1DM, certified nurses, anthropologists and a bioethics expert. This expertize helped clarify doubts and the contextualization of the findings.

The characteristics of individual studies were collected in a table specifically designed for this review. The table includes the first author, year of publication, country, aim of the study, qualitative study design, sample, setting and methodological limitations of the study. The CASPe checklist was used as a critical appraisal tool to assess the methodological quality of the studies.^22^

## 3 RESULTS

Eighty‐four were selected citations which included 21 duplicates. Sixty‐three articles were screened by title and abstract and 39 references were assessed for eligibility by reading their full texts. Six additional references were found in those full‐text articles. Ten references were finally included. Figure 1 shows the PRISMA flow diagram for the selection of the studies.

The characteristics of the included studies are shown in Table 4. Studies were variable in terms of setting, aim, qualitative design and population. All studies were set in high‐income countries. Six studies took place in the United Kingdom, two in Sweden and one in Australia and one in the United States of America. All studies used interviews and/or focus groups as data collection techniques. The population included women with T1DM at childbearing age, nonpregnant, pregnant or those already had given birth. Most studies included none or few women of diverse ethnicity^25^, ^26^, ^27^, ^28^, ^29^, ^30^, ^31^, ^32^ or did not collect ethnicity date^23^, ^24^ and there was little information about other social determinants of health. The quality assessments of the studies are summarized in Supporting Information: File S3.

**Table 4:** Characteristics of the studies.

First author, year and country	Aim of the study	Qualitative study design	Sample (*n*)	Setting	Methodological limitations (CASPe)
Adolfsson, 2012^23^ Sweden	To analyze the experiences of pregnant women and mothers with T1DM using a prototype of the MODIAB‐web by reporting on the communication between the women, their midwives, and the researchers and data programmers as part of this pilot programme.	Focus group.	Women with with T1DM who had also experienced pregnancy, breastfeeding and nurturing of children (*n* = 3).	A medium‐sized hospital.	The design and use of qualitative methodology is partially adequate. The sample is very small and the data collection technique is not described accurately.
Berg, 2009^24^ Sweden	To explore the need and experience of professional support during pregnancy and childbirth in women with type 1 diabetes.	Focus group and individual interviews.	Women with T1DM who have had one or more children (*n* = 23).	Residents in the western region of Sweden.	The design and use of qualitative methodology is adequate. The analysis of the data is not described in depth.
Earle, 2017^25^ UK	1. To understand why women with DM of childbearing age access or not preconception care services, to inform the services. 2. To investigate the views of staff and stakeholders to explore the current and future provision of preconception care.	Interviews.	Women of childbearing age with pre‐existing DM (type 1 and 2) (*n* = 12). The results are explained separately.	Primary care, England	The design and use of qualitative methodology is adequate.
Griffiths, 2008^26^ UK	To explore women's accounts of their journeys to become pregnant while living with with T1DM.	Semi‐structured interviews.	Women living with T1DM between 20 and 30 weeks of pregnancy and a normal pregnancy ultrasound scan (*n* = 15).	Antenatal clinics in the West Midlands in England.	The design and use of qualitative methodology is adequate.
King, 2007^27^ Australia	To explore the women's experience of their preconception preparation and pregnancy, and to describe the women's engagement with health care providers during this period.	In‐depth interviews.	Women with T1DM who had given birth in the previous 12 months, English‐speaking, residing in regional and rural Australia, and over 18 years of age (*n* = 6).	Australia.	The design and use of qualitative methodology is adequate. The analysis of the data is not described in depth.
McCorry,^28^ 2012 UK	To explore attitudes towards pregnancy planning and preconception care seeking among women with T1DM.	In‐depth semi‐structured interviews.	Nonpregnant women with T1DM (*n* = 14).	Residents in the South Eastern Health and Social Care Trust area in Northern Ireland	The design and use of qualitative methodology is adequate.
McGrath, 2017^29^ USA	To study the pregnancy experiences of women with T1DM and to seek their views on what health care providers could do to help them have a healthy pregnancy.	Semi‐structured interviews.	Women with T1DM who were pregnant at the time of the study or who had been pregnant previously (*n* = 10).	Recruitment through Facebook.	The participant's selection strategy is partially congruent with the research question and the method used, since the recruitment was through the first author's contact network on Facebook. The results are partially applicable, taking into account the limitations of the sample and lack of diversity.
Richmond, 2009^30^ UK	The aims of the study were: to identify changes in glycemic control in women with T1DM during and after pregnancy and explore the feelings of the women before and after childbirth; to identify experiences attributed to the changes in their glycemic control; to identify issues which would contribute to the future planning of care for women with T1DM embarking on pregnancy.	Interviews.	Women with T1DM attending the joint obstetric/diabetic clinic in a district general hospital between October 2001 and April 2004 (*n* = 11).	Obstetric/diabetic clinic in a district general hospital.	The design and use of qualitative methodology is adequate, but the type of interview conducted is not specified.
Woolley, 2015^31^ UK	To explore women's perceptions and experiences of being pregnant and having pre‐existing T1DM, and to assess their physical, social, psychological, emotional and educational needs during their transition to motherhood.	Interviews with open‐ended questions.	Women with T1DM in their first pregnancy (*n* = 7).	Antenatal medical disorders clinic of a district general maternity hospital in England.	The design and use of qualitative methodology is adequate.
Wotherspoon, 2017^32^ UK	To explore knowledge of pre‐eclampsia and opinions on possible screening tests for pre‐eclampsia in women with T1DM.	Semi‐structured interviews.	Women with T1DM planning pregnancy, pregnant or postpartum with experience of pre‐eclampsia (*n* = 11).	Prepregnancy care clinic or the joint antenatal‐metabolic clinic within the Belfast Health and Social Care Trust in Northern Ireland.	The design and use of qualitative methodology is adequate. The research results are partially applicable, due to the homogeneity of the sample.

Abbreviations: DM, diabetes mellitus; T1DM, type 1 diabetes mellitus.

### 3.1 Experiences of women with T1DM regarding pregnancy and its planning

Pregnancy with diabetes requires hard work to manage optimum glycemic levels, which includes feelings of pressure, mental effort and anxiety due to the risk to the foetus.^27^, ^30^, ^31^ But women with T1DM and children also consider pregnancy doable and worth the effort.^29^ The desire to get pregnant and have children is full of fears and anxiety for many women with T1DM.^28^ Pregnancy planning is important for women with T1DM who want to become pregnant and this can take them anywhere from a few days to years.^27^, ^28^, ^31^

These experiences are expressed in the following quotations:

I just think the lines that I am trying to keep my blood sugars between are much tighter, so it is a lot of hard work. (McGrath, 2017)

I know because I'm pregnant I've got to look after myself more because I don't want anything to happen to the baby because of it… because of me not looking after me self… (Richmond, 2009)

It's really important for me to get better [metabolic] control because of the baby. I know I haven't been good. I am trying really hard!. (Richmond, 2009)

Plan, plan, plan! And if you do not want kids, take proper precautions. There is enough stuff out there for your pregnancy to be planned. (McCorry, 2012)

### 3.2 Acceptability of preconception and antenatal care

Although there are mixed views about the experience of preconception and antenatal care, it may be acceptable to the majority of women with T1DM; however, in some cases, receiving this counselling can trigger fear and anxiety which may become a burden for these women.^23^, ^24^, ^25^, ^26^, ^31^ There is an important variability about what women consider adequate information regarding diabetes and pregnancy; some women demand more information, others prefer not to know many details because this can generate anxiety and or feelings of being overwhelmed.^25^, ^27^, ^28^, ^32^ Timing of information is also important, since women sometimes receive a lot of information at a prenatal appointment, and they consider that it may be more helpful to receive such information further into their pregnancy.^32^

Individualized care, having a comfortable and trusting relationship with a professional who can identify their individual needs, including the complexity of diabetes management, can improve acceptability.^25^, ^27^, ^28^, ^29^, ^31^ Support from the antenatal unit and a diabetes midwife provides women with confidence about the pregnancy.^23^, ^24^, ^25^, ^26^, ^31^ Women with T1DM receive spontaneous pregnancy advice from an early age. These early tips are welcome in helping plan for a healthy pregnancy.^23^ However, on many occasions, women with T1DM receive discouraging or fear‐mongering messages from both health professionals and other people. These experiences discourage them both from becoming pregnant and from attending prepregnancy consultations.^25^, ^27^, ^28^, ^29^

The following vignettes allow us to illustrate the above:

… before you're pregnant they give you information about … what high blood sugar could do to a baby and it's not nice reading; I should imagine it scares a lot of people to keep their blood sugars good. (Wooley, 2015)

We'd gone to the hospital, and we'd had prepregnancy counselling … which wasn't very positive … it was a very, very negative experience. We came away from there and I was very upset and [husband] was quite upset too and then we started talking about adoption and fostering. We were filled with dread really about the consequences of getting pregnant—for me and for the baby. That was November‐time and then in the January I sort of thought I'm never going to rest if I don't … you know … if I'm not getting pregnant myself, so let's just … I'm going to look after myself and let's just go for it. (Griffith, 2008)

I didn't know before preconception. Oh, I had an abundance of leaflets to look at. […] You know these little lives could be deformed just ‘cos you can't be bothered to look after yourself and you could have done things wrong, couldn't you?’. (Earle, 2017)

… but it was mainly just for bloods and how was your control. There was never really anything else. (Wotherspoon, 2017)

… he just told us that it had to be checked every so many weeks, every 6 weeks at least, but he didn't really go into detail why. (Wotherspoon, 2017)

Interviewer: Erm what do you think helps facilitate the care? Lilly: The staff, the people, they are just lovely. Nothing is too much trouble. (Earle, 2017)

The Diabetes Team are always at the end of the phone … that gives us the confidence … if anything crops up, we call them, they help us. (Wooley, 2015)

The specialist there in the hospital he was great … really good, really laid back, said ‘5.9 fabulous, you know your stuff if you need anything come and see me’… but when we had a relieving doctor it was really difficult because I would always be confronted with a doctor saying oh you can expect to have a still birth, … you're going to have a handicapped child … oh lovely things … (King, 2007)

[…] [M]y care felt much more like a conversation, it felt more collaborative. I didn't go in and was told I should do this, it felt much more like what's your view, what are you doing, shall we try this? What do you think on it? That kind of conversation. (Earle, 2017)

As a child when I was diagnosed, at that time, I was told that I may never have children and that if I was to have children that perhaps the last 3 months of pregnancy would be spent in Melbourne [major city], in bed and restricted and things like that, so having children was always a scary, scary thing for me. (King, 2007)

### 3.3 Feasibility and implementation considerations for preconception or antenatal care

Lack of awareness about the importance of preconception care are some of the barriers for feasibility. Not all women with T1DM are aware of the risks of pregnancy. Awareness is an incentive to attend antenatal care.^25^, ^28^ The most common reason for women's attendance was for a referral.^25^ In addition, women who had lost their babies in previous pregnancies sought preconceptual help for a new pregnancy.^30^ Another barrier for feasibility might be the lack of support from their peers. Women who had not received support from their peers said they wished they had. Thus, access to antenatal care may be improved through contact with other women in the same situation, and sharing experiences, which seems to be important for women.^23^, ^24^, ^29^ Health care professionals working with young diabetic patients should contemplate hosting discussion groups for mothers and nulliparous women.^29^

The third barrier identified is the need for specialized, qualified or trained health care professionals in both diabetes and pregnancy. Health professionals who are not specialized in diabetes and care for women with T1DM during pregnancy do not always have the necessary skills or training to help these women properly with their pregnancy management.^24^, ^27^

Finally, the continuity of care, coordination between health professionals and services, a more holistic approach in identifying individual needs and recognizing patients as experts in their own condition can alleviate women's frustrations with the medical model of care is a key aspect for women.^24^, ^25^, ^28^, ^31^, ^32^ The midwife's role is considered fundamental in the provision of normalized care for pregnant women.^31^

These are some of the quotations that illustrate these ideas:

… sure, those who work with diabetes and pregnancy know the facts of how diabetes works, but they can never understand the feelings that are involved, how you make it work in your everyday life, they can only provide tips about how others deal with it, it's easier to talk to someone who is actually in the exact same situation. (Adolfsson, 2012)

Basically, what I tell them is that I felt the same way you do now when I saw other type‐1 women pregnant. It is hard work, and you really have to plan, but it is also doable, especially if you have the right support, it's totally doable. (McGrath, 2017)

Telling my story … I think was a big thing. Every time I met a new professional I had to explain. An’ it irritated me after a while,'cos I thought ‘Read the notes before you walk into the room to see me’. (Wooley, 2015)

The fact that the diabetes team are coming over here, to the Maternity … it makes this a more ‘normal’ pregnancy, although I'm here every week about me diabetes … (Wooley, 2015)

I went into my ordinary antenatal clinic in my hometown, for ordinary antenatal care, because you didn't get much of that here (at the hospital). So I had the midwife there for another type of support, advice and other ordinary things. I came here more to see doctors and get checked up with scans and things like that. So, it was very divided. (Berg, 2009)

### 3.4 Equity and preconception or antenatal care accessibility

The included studies did not explore the specificities of the experiences of women from ethnically, educational or socioeconomically diverse backgrounds. Access was the main topic related to equity. Accessibility to pre‐conception clinics on a regular basis has barriers, such as the adapting working hours, being unable to park or the unpredictably long waits and limited levels of experience with diabetes and pregnancy, particularly in rural areas, were perceived. Referral seems to improve accessibility.^25^, ^27^

These ideas can be followed through these quotations:

There was a lot of fear and I guess that's because of a combination of a lack of knowledge, lack of resources, lack of networks and fear of litigation. (King, 2007)

Basically, he's about the only endocrinologist on the Coast, you just don't have any choice here … I've actually only seen him twice … I would like to have someone to be talking to more regularly about the diabetes. (King, 2007)

## 4 DISCUSSION

The findings of the present study show the variety of experiences that women with T1DM have in relation to antenatal care. Thus, the main results confirm that (1) preconception and antenatal care might be acceptable to the majority of women with T1DM, with some exceptions; (2) antenatal care may improve with individualized care, continuity of care, coordination within the health care system, and peer support; and (3) to increase equitability, preconception care needs to be improved in rural areas. Although in a previous review Earle et al.^25^ explored views on the provision of, and facilitators of and barriers to the uptake of, preconception care through qualitative research, the present study is the first QES analyzing acceptability, feasibility and equity addressing a research gap. In a recent paper aimed at discussing solutions to improve antenatal care quality, access and delivery, the authors stated that more attention should be paid to a fuller understanding from the user's perspective, that should be inclusive, and that this could help to reduce some of the barriers to quality care.^33^

Experiences of care should be taken into account when developing CPGs and making evidence‐based recommendations to support a better implementation of these recommendations. In this regard, a QES can provide decision‐makers with additional evidence to improve their understanding of the complexity of the interventions, contextual variations and further understanding of values, attitudes and experiences of those who receive the interventions or who implement them.^16^ In line with person‐centred care frameworks,^34^, ^35^ the results here point to the importance of a more holistic approach, individualized care, continuity of care, better coordination between health professionals and services and one in which pregnant T1DM women can be recognized as experts in their diabetes, while having more focus on the pregnancy itself. In diabetes care, personalized care planning has a proven small positive effect in measured glycated haemoglobin (HbA1c), when compared to usual care.^36^ Person‐centred care can be implemented by integrating the elicitation of personalized goals, preparing a care plan that includes the care delivery process and the monitoring of the goal attainment.^34^ To ensure continuity of care, professional and interdisciplinary cooperation at the micro, meso and macro levels needs to be enhanced.^37^ In conception care for T1DM women, the role of the midwife is central but needs to be coupled with specialized diabetes care.

The feasibility of person‐centred antenatal care for women with T1DM requires the promotion of awareness of the need for such care and the planned interventions, improved pathways for patients and training for professionals. A full assessment of feasibility could be complemented by a review of studies that consider the perspectives of the different health professionals (midwives, diabetes specialists and others) and settings involved (primary/specialist care or antenatal clinics), and the costs of the interventions. Health care professionals may need training and communication skills to provide person‐centred T1DM antenatal care.^38^ A study evaluating a regional antenatal care programme may show savings when the excess costs of adverse pregnancy outcomes are taken into account_._ ^39^

Finally, further research on equity is needed as the included studies did not reflect the impact of social determinants on ethnic minorities or socioeconomically deprived women who are more likely to experience T1DM and have worse obstetric outcomes.^40^, ^41^, ^42^ Recruitment seems to be an important consideration as, even in studies with a focus on ethnic diversity, participants ended up being mostly white.^25^, ^43^ Nevertheless, the need to invest in improving accessibility to professional care with experience in diabetes and pregnancy in rural areas is clear. In this sense, a recent systematic review exploring rural health care delivery and maternal and infant outcomes for diabetes in pregnancy.^44^ shows a gap in published research in the matter as it identified only two studies on such interventions.^45^, ^46^ Both models proposed a specific model of care adapted to rural areas. Only one, Murfet et al.^45^ reported an improvement in neonatal outcomes and did not increase the number of specialist referrals by forming a multidisciplinary team coordinated by a nursing practitioner which included a dietitian, diabetes educator, obstetrician and antenatal nurse.

## 5 LIMITATIONS

This study has some limitations. First, only studies in Spanish and English were included, which may have excluded relevant works in other languages and contexts. Most of the articles included were from anglo‐saxon or European countries. Nevertheless, local stakeholders such as patients, patient organizations or health care professionals participating in the development of the CPG discussed and contrasted the findings to adapt the recommendations to the local context. Second, due to the need for rapid GPC recommendations, the search was restricted to the period from 2011 to 2021, and no grey literature was included, which may have excluded some relevant articles. However, due to their relevance, articles before 2011 were added after checking the references. Third, it was not possible to register the protocol of the review due to a pressing deadline for the CPG, but a version in Spanish can be provided.

## 6 CONCLUSIONS

The findings here show that preconception care may be acceptable to the majority of women with T1DM, although the importance of individualized care and trusting relationships with the professionals to improve acceptability should be mentioned. Continuity of care, coordination between health professionals and services and a more holistic approach are key aspects for women that need to be considered for more feasible antenatal care. Finally, in rural areas, limited levels of experience with diabetes and pregnancy were perceived, which can mean inequitable access. Antenatal care is highly variable and dependent on many factors, such as the geographical area or the professionals' training. More protocols are needed to support women with T1DM in prepregnancy interventions and during pregnancy, taking into account issues of acceptability, feasibility and equity.

## CONFLICT OF INTEREST STATEMENT

The authors declare no conflict of interest.

## Supporting information

Additional file 1 –Enhancing transparency in reporting the synthesis of qualitative research: ENTREQ Checklist.

Additional file 2 – Search Strategies.

Additional file 3 ‐ Summary of the Checklist Critical Appraisal Skillls Programme in Spanish (CASPe).

## Data Availability

The data that support the findings of this study are available on request from the corresponding author. The data are not publicly available due to privacy or ethical restrictions.
