# Supplementary material for: Perspectives of women living with type 1 diabetes regarding preconception and antenatal care: A qualitative evidence synthesis
Source: Health Expect. 2023 Nov 1;27(1):e13876. doi: 10.1111/hex.13876 (PMC10726142; doi:10.1111/hex.13876)
Supplement: Supplementary file 2 — Additional file 2 – Search Strategies. [file HEX-27-e13876-s001.docx]

**Additional File 2: Search Strategies**

**Medline**

| Ovid MEDLINE(R) ALL <1946 to April 08, 2021> | | |
| --- | --- | --- |
| 1 | Diabetes Mellitus, Type 1/ | 77416 |
| 2 | exp Diabetic Ketoacidosis/ | 6620 |
| 3 | (diabet$ adj3 (britt$ or juvenil$ or pediatric or paediatric or child$ or early or keto$ or labil$ or acidos$ or autoimmun$ or auto immun$ or sudden onset or typ$ 1 or typ$ I)).ti,ab,hw. | 115264 |
| 4 | (insulin depend$ or insulindepend$ or insulin-depend$).ti,ab,kw. | 29650 |
| 5 | (IDDM or T1DM or T1D or dm1 or dm 1 or dmt1 or dm t1 or t1 dm).ti,ab,kw. | 23511 |
| 6 | 1 or 2 or 3 or 4 or 5 | 134622 |
| 7 | exp Diabetes Insipidus/ | 7970 |
| 8 | diabet$ insipidus.tw. | 8817 |
| 9 | 7 or 8 | 11077 |
| 10 | 6 not 9 | 134047 |
| 11 | Preconception Care/ | 2416 |
| 12 | preconcept* care.ti,ab. | 684 |
| 13 | Maternal Health Services/ | 14372 |
| 14 | (Preconcept* or "Pre‐concept*" or Prepregnan* or "Pre‐pregnan*").ti,ab. | 9252 |
| 15 | ((pregnan$ or conception or family) adj3 plan$).ti,ab. | 30935 |
| 16 | ((Preconcept$ or "Pre‐concept$" or Prepregnan$ or "Pre‐pregnan$") adj2 (care or counsel$ or advice$ or advise or inform$)).ti,ab. | 1542 |
| 17 | 11 or 12 or 13 or 14 or 15 or 16 | 54202 |
| 18 | 10 and 17 | 535 |
| 19 | limit 18 to yr="2011 -Current" | 261 |
| 20 | limit 19 to (english language or spanish) | 246 |
| 21 | ((("semi-structured" or semistructured or unstructured or informal or "in-depth" or indepth or "face-to-face" or structured or guide) adj3 (interview* or discussion* or questionnaire*)) or (focus group* or qualitative or ethnograph* or fieldwork or "field work" or "key informant")).ti,ab. or interviews as topic/ or focus groups/ or narration/ or qualitative research/ | 419707 |
| 22 | 20 and 21 | 16 |

**Embase**

| **# 16** | [**43**](https://apps.webofknowledge.com/summary.do?product=UA&doc=1&qid=55&SID=F2UUzISEhGTKfTXes2N&search_mode=AdvancedSearch&update_back2search_link_param=yes) | **#15** |
| --- | --- | --- |
|  |  | ***Bases de datos= WOS, CCC, DIIDW, KJD, MEDLINE, RSCI, SCIELO Período de tiempo=2011-2021*** |
|  |  | ***Idioma de búsqueda=Auto*** |
| **# 15** | [**66**](https://apps.webofknowledge.com/summary.do?product=UA&doc=1&qid=54&SID=F2UUzISEhGTKfTXes2N&search_mode=CombineSearches&update_back2search_link_param=yes) | **#14 AND #13** |
|  |  | ***Bases de datos= WOS, CCC, DIIDW, KJD, MEDLINE, RSCI, SCIELO Período de tiempo=1900-2021*** |
|  |  | ***Idioma de búsqueda=Auto*** |
| **# 14** | [**3.635.993**](https://apps.webofknowledge.com/summary.do?product=UA&doc=1&qid=51&SID=F2UUzISEhGTKfTXes2N&search_mode=AdvancedSearch&update_back2search_link_param=yes) | **TS=(qualitative OR ethnol* OR ethnog* OR ethnonurs* OR emic OR etic OR leininger OR noblit OR "field note*" OR "field record*" OR fieldnote* OR "field stud*" or "participant observ*" OR "participant observation*" OR hermaneutic* OR phenomenolog* OR "lived experience*" OR heidegger* OR husserl* OR "merleau-pont*" OR colaizzi OR giorgi OR ricoeur OR spiegelberg OR "van kaam" OR "van manen" OR "grounded theory" OR "constant compar*" OR "theoretical sampl*" OR glaser AND strauss OR "content analy*" OR "thematic analy*" OR narrative* OR "unstructured categor*" OR "structured categor*" OR "unstructured interview*" OR "semi-structured interview*" OR "maximum variation*" OR snowball OR audio* OR tape* OR video* OR metasynthes* OR "meta-synthes*" OR metasummar* OR "meta- summar*" OR metastud* OR "meta-stud*" OR "meta-ethnograph*" OR metaethnog* OR "meta-narrative*" OR metanarrat* OR " meta-interpretation*" OR metainterpret* OR "qualitative meta-analy*" OR "qualitative metaanaly*" OR "qualitative etanaly*" OR "purposive sampl*" OR "action research" OR "focus group*" or photovoice or "photo voice" or "mixed method*")** |
|  |  | ***Bases de datos= WOS, CCC, DIIDW, KJD, MEDLINE, RSCI, SCIELO Período de tiempo=1900-2021*** |
|  |  | ***Idioma de búsqueda=Auto*** |
| **# 13** | [**1.114**](https://apps.webofknowledge.com/summary.do?product=UA&doc=1&qid=49&SID=F2UUzISEhGTKfTXes2N&search_mode=AdvancedSearch&update_back2search_link_param=yes) | **#12** |
|  |  | ***Bases de datos= WOS, CCC, DIIDW, KJD, MEDLINE, RSCI, SCIELO Período de tiempo=1900-2021*** |
|  |  | ***Idioma de búsqueda=Auto*** |
| **# 12** | [**1.114**](https://apps.webofknowledge.com/summary.do?product=UA&doc=1&qid=48&SID=F2UUzISEhGTKfTXes2N&search_mode=CombineSearches&update_back2search_link_param=yes) | **#11 AND #6** |
|  |  | ***Bases de datos= WOS, CCC, DIIDW, KJD, MEDLINE, RSCI, SCIELO Período de tiempo=1900-2021*** |
|  |  | ***Idioma de búsqueda=Auto*** |
| **# 11** | [**109.792**](https://apps.webofknowledge.com/summary.do?product=UA&doc=1&qid=47&SID=F2UUzISEhGTKfTXes2N&search_mode=CombineSearches&update_back2search_link_param=yes) | **#10 OR #9 OR #8 OR #7** |
|  |  | ***Bases de datos= WOS, CCC, DIIDW, KJD, MEDLINE, RSCI, SCIELO Período de tiempo=1900-2021*** |
|  |  | ***Idioma de búsqueda=Auto*** |
| **# 10** | [**4.708**](https://apps.webofknowledge.com/summary.do?product=UA&doc=1&qid=46&SID=F2UUzISEhGTKfTXes2N&search_mode=AdvancedSearch&update_back2search_link_param=yes) | **TS= ((Preconcept* or "Pre‐concept*" or Prepregnan* or "Pre‐pregnan*") NEAR/2 (care or counsel* or advice* or advise or inform*) )** |
|  |  | ***Bases de datos= WOS, CCC, DIIDW, KJD, MEDLINE, RSCI, SCIELO Período de tiempo=1900-2021*** |
|  |  | ***Idioma de búsqueda=Auto*** |
| **# 9** | [**88.836**](https://apps.webofknowledge.com/summary.do?product=UA&doc=1&qid=45&SID=F2UUzISEhGTKfTXes2N&search_mode=AdvancedSearch&update_back2search_link_param=yes) | **TS= ((pregnan* or conception or family) NEAR/3 plan*)** |
|  |  | ***Bases de datos= WOS, CCC, DIIDW, KJD, MEDLINE, RSCI, SCIELO Período de tiempo=1900-2021*** |
|  |  | ***Idioma de búsqueda=Auto*** |
| **# 8** | [**22.278**](https://apps.webofknowledge.com/summary.do?product=UA&doc=1&qid=44&SID=F2UUzISEhGTKfTXes2N&search_mode=AdvancedSearch&update_back2search_link_param=yes) | **TS= (Preconcept* or "Pre‐concept*" or Prepregnan* or "Pre‐pregnan*")** |
|  |  | ***Bases de datos= WOS, CCC, DIIDW, KJD, MEDLINE, RSCI, SCIELO Período de tiempo=1900-2021*** |
|  |  | ***Idioma de búsqueda=Auto*** |
| **# 7** | [**4.747**](https://apps.webofknowledge.com/summary.do?product=UA&doc=1&qid=43&SID=F2UUzISEhGTKfTXes2N&search_mode=AdvancedSearch&update_back2search_link_param=yes) | **TS= (preconcept* care)** |
|  |  | ***Bases de datos= WOS, CCC, DIIDW, KJD, MEDLINE, RSCI, SCIELO Período de tiempo=1900-2021*** |
|  |  | ***Idioma de búsqueda=Auto*** |
| **# 6** | [**317.866**](https://apps.webofknowledge.com/summary.do?product=UA&doc=1&qid=42&SID=F2UUzISEhGTKfTXes2N&search_mode=AdvancedSearch&update_back2search_link_param=yes) | **#4 not #5** |
|  |  | ***Bases de datos= WOS, CCC, DIIDW, KJD, MEDLINE, RSCI, SCIELO Período de tiempo=1900-2021*** |
|  |  | ***Idioma de búsqueda=Auto*** |
| **# 5** | [**15.978**](https://apps.webofknowledge.com/summary.do?product=UA&doc=1&qid=41&SID=F2UUzISEhGTKfTXes2N&search_mode=AdvancedSearch&update_back2search_link_param=yes) | **TS= diabet* insipidus** |
|  |  | ***Bases de datos= WOS, CCC, DIIDW, KJD, MEDLINE, RSCI, SCIELO Período de tiempo=1900-2021*** |
|  |  | ***Idioma de búsqueda=Auto*** |
| **# 4** | [**318.465**](https://apps.webofknowledge.com/summary.do?product=UA&doc=1&qid=40&SID=F2UUzISEhGTKfTXes2N&search_mode=CombineSearches&update_back2search_link_param=yes) | **#3 OR #2 OR #1** |
|  |  | ***Bases de datos= WOS, CCC, DIIDW, KJD, MEDLINE, RSCI, SCIELO Período de tiempo=1900-2021*** |
|  |  | ***Idioma de búsqueda=Auto*** |
| **# 3** | [**38.753**](https://apps.webofknowledge.com/summary.do?product=UA&doc=1&qid=39&SID=F2UUzISEhGTKfTXes2N&search_mode=AdvancedSearch&update_back2search_link_param=yes) | **TS=(dm1 or "dm 1" or dmt1 or "dm t1" or t1dm or "t1 dm" or t1d or iddm)** |
|  |  | ***Bases de datos= WOS, CCC, DIIDW, KJD, MEDLINE, RSCI, SCIELO Período de tiempo=1900-2021*** |
|  |  | ***Idioma de búsqueda=Auto*** |
| **# 2** | [**195.960**](https://apps.webofknowledge.com/summary.do?product=UA&doc=1&qid=38&SID=F2UUzISEhGTKfTXes2N&search_mode=AdvancedSearch&update_back2search_link_param=yes) | **TS=((insulin* NEAR/2 depend*) or insulindepend*)** |
|  |  | ***Bases de datos= WOS, CCC, DIIDW, KJD, MEDLINE, RSCI, SCIELO Período de tiempo=1900-2021*** |
|  |  | ***Idioma de búsqueda=Auto*** |
| **# 1** | [**172.396**](https://apps.webofknowledge.com/summary.do?product=UA&doc=1&qid=37&SID=F2UUzISEhGTKfTXes2N&search_mode=AdvancedSearch&update_back2search_link_param=yes) | **TS=(diabet* NEAR/3 ("'type 1" OR "type i" OR britt* OR juvenil* OR pediatric OR paediatric OR early OR keto* OR labil* OR acidos* OR autoimmun* OR "auto immun*" OR "sudden onset") )** |
|  |  | ***Bases de datos= WOS, CCC, DIIDW, KJD, MEDLINE, RSCI, SCIELO Período de tiempo=1900-2021*** |
|  |  | ***Idioma de búsqueda=Auto*** |

**WoS**

| Embase Session Results |
| --- |
| **No.** |
| **Query** |
| **Results** |
| **25** |
| **#22** |
| **#20** AND **#21** |
| **192,44** |
| **#21** |
| ((((**'semi structured'** OR **semistructured** OR **unstructured** OR **informal** OR **'in-depth'** OR **indepth** OR **'face-to-face'** OR **structured** OR **guide**) NEAR/3 (**interview*** OR **discussion*** OR **questionnaire***)):ti,de) OR (**focus**:ti,de AND **group***:ti,de) OR **qualitative**:ti,de OR **ethnograph***:ti,de OR **fieldwork**:ti,de OR **'field work'**:ti,de OR **'key informant'**:ti,de OR **'qualitative research'**/exp) AND ([english]/lim OR [spanish]/lim) AND (**'article'**/it OR **'letter'**/it OR **'note'**/it OR **'review'**/it OR **'article in press'**/it) |
| **1,275** |
| **#20** |
| **#10** AND **#17** AND [2011-2021]/py AND ([english]/lim OR [spanish]/lim) |
| **1,318** |
| **#19** |
| **#10** AND **#17** AND [2011-2021]/py |
| **2,068** |
| **#18** |
| **#10** AND **#17** |
| **93,041** |
| **#17** |
| **#11** OR **#12** OR **#13** OR **#14** OR **#15** OR **#16** |
| **2,306** |
| **#16** |
| ((**preconcept*** OR **'pre‐concept*'** OR **prepregnan*** OR **'pre‐pregnan*'**) NEAR/2 (**care** OR **counsel*** OR **advice*** OR **advise** OR **inform***)):ti,ab |
| **33,171** |
| **#15** |
| ((**pregnan*** OR **conception** OR **family**) NEAR/3 **plan***):ti,ab |
| **12,994** |
| **#14** |
| **preconcept***:ti,ab OR **'pre‐concept*'**:ti,ab OR **prepregnan***:ti,ab OR **'pre‐pregnan*'**:ti,ab |
| **50,101** |
| **#13** |
| **'maternal care'**/exp |
| **933** |
| **#12** |
| **'preconcept* care'**:ti,ab |
| **2,028** |
| **#11** |
| **'prepregnancy care'**/exp |
| **434,026** |
| **#10** |
| **#6** NOT **#9** |
| **16,298** |
| **#9** |
| **#7** OR **#8** |
| **15,752** |
| **#8** |
| **'diabet* insipidus'**:ti,ab,de |
| **14,89** |
| **#7** |
| **'diabetes insipidus'**/exp |
| **408,209** |
| **#6** |
| **#1** OR **#2** OR **#3** OR **#4** OR **#5** |
| **37,02** |
| **#5** |
| **iddm**:ti,ab,de OR **t1dm**:ti,ab,de OR **t1d**:ti,ab,de OR **dm1**:ti,ab,de OR **'dm 1'**:ti,ab,de OR **dmt1**:ti,ab,de OR **'dm t1'**:ti,ab,de OR **'t1 dm'**:ti,ab,de |
| **355,947** |
| **#4** |
| ((**insulin*** NEAR/2 **depend***):ti,ab,de) OR **insulindepend***:ti,ab,de |
| **128,326** |
| **#3** |
| (**diabet*** NEAR/3 (**britt*** OR **juvenil*** OR **pediatric** OR **paediatric** OR **child*** OR **early** OR **keto*** OR **labil*** OR **acidos*** OR **autoimmun*** OR **'auto immun*'** OR **'sudden onset'** OR **'type 1'** OR **'type i'**)):ti,ab,de |
| **12,509** |
| **#2** |
| **'diabetic ketoacidosis'**/exp |
| **344,988** |
| **#1** |
| **'insulin dependent diabetes mellitus'**/exp OR **'insulin dependent diabetes mellitus'** |
