# Supplementary material for: Perspectives of women living with type 1 diabetes regarding preconception and antenatal care: A qualitative evidence synthesis
Source: Health Expect. 2023 Nov 1;27(1):e13876. doi: 10.1111/hex.13876 (PMC10726142; doi:10.1111/hex.13876)
Supplement: Supplementary file 3 — Additional file 3 ‐ Summary of the Checklist Critical Appraisal Skillls Programme in Spanish (CASPe). [file HEX-27-e13876-s002.doc]

| **Summary of the Checklist Critical Appraisal Skillls Programme in Spanish (CASPe)** | | | | | | | | | | | |
| --- | --- | --- | --- | --- | --- | --- | --- | --- | --- | --- | --- |
|  | **Adolfsson 2012** | **Berg 2009** | **Earle, 2017** | **Griffiths 2008** | **King, 2009** | **McCorry, 2012** | **McGrath, 2017** | **Richmond**  **2009** | **Woolley 2015** | **Wotherspoon, 2017** | |
| 1. Was there a clear  statement of the aims of the research? | Yes | Yes | Yes | Yes | Yes | Yes | Yes | Yes | Yes | Yes | |
| 2. Is a qualitative  methodology  appropriate? | Yes | Yes | Yes | Yes | Yes | Yes | Yes | Yes | Yes | Yes | |
| 3. Was the research  design appropriate to address the aims of the research? | Partially | Yes | Yes | Yes | Yes | Yes | Yes | Yes | Yes | Yes | |
| 4. Was the recruitment strategy appropriate to the aims of the  research? | Yes | Yes | Yes | Yes | Yes | Yes | Partially, recruitment through author’s contacts in Facebook | Yes | Yes | Yes | |
| 5. Was the data collected in  a way that addressed the  research issue? | Partially | Yes | Yes | Yes | Yes | Yes | Yes | The type of interview is not specified | Yes | Yes | |
| 6. Has the relationship between researcher and participants been adequately considered? | No | Yes | No | No | Yes | Yes | No | No | No | No | |
| 7. Have ethical issues been taken into consideration? | Yes | Yes | Yes | Yes | Yes | Yes | Yes | Can’t tell | Yes | Yes | |
| 8. Was the data analysis  sufficiently rigorous? | It is not well described | It is not well described | Yes | Yes | It is not well described | Yes | Yes | Yes | Yes | Yes |  |
| 9. Is there a clear statement  of findings? | Yes | Yes | Yes | Yes | Yes | Yes | Yes | Yes | Yes | Yes |  |
| 10. How valuable is the research? | Partially, due to the aim of the study | Yes | Yes | Yes | Yes | Yes | Partially, due to sample limitations | Yes | Yes | Partially, due to lack of diversity |  |
